# Methyl 3-dehydr­oxy-3-oxoursolate

**DOI:** 10.1107/S1600536809030669

**Published:** 2009-08-08

**Authors:** Khalijah Awang, Nor Hayati Abdullah, Noel F. Thomas, Seik Weng Ng

**Affiliations:** a Department of Chemistry, University of Malaya, 50603 Kuala Lumpur, Malaysia; b Medicinal Plant Division, Forest Research Institute Malaysia, 52100 Kepong, Selangor Darul Ehsan, Malaysia

## Abstract

Four of the five six-membered rings of the title penta­cylic triterpene, C_31_H_48_O_3_, adopt chair conformations; the fifth, which has a C=C double bond, adopts an approximate envelope conformation.

## Related literature

The structure was previously refined to an *R*-index of 0.043 but atomic coordinates were not published. The reported room-temperature cell [8.109 (1), 8.618 (1), 39.148 (1) Å] is slightly larger; see: de Vivar *et al.* (1985 ▶). For the synthesis, see: Honda *et al.* (1997 ▶); Ma *et al.* (2005 ▶); Zhao *et al.* (2007 ▶).
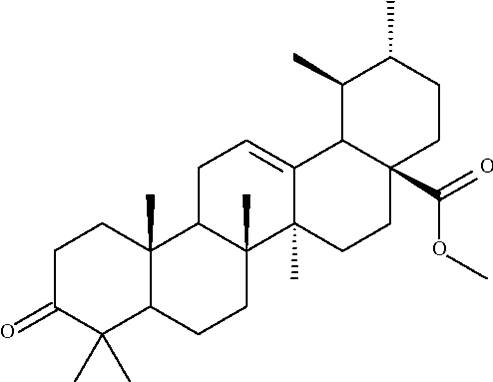


## Experimental

### 

#### Crystal data

C_31_H_48_O_3_
*M* *_r_* = 468.69
Orthorhombic, 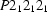

*a* = 8.0298 (2) Å
*b* = 8.4775 (2) Å
*c* = 39.0492 (7) Å
*V* = 2658.2 (1) Å^3^
*Z* = 4
Mo *K*α radiation
μ = 0.07 mm^−1^
*T* = 100 K
0.25 × 0.15 × 0.10 mm

#### Data collection

Bruker SMART APEX diffractometer
Absorption correction: none
18588 measured reflections
3500 independent reflections
3188 reflections with *I* > 2σ(*I*)
*R*_int_ = 0.041

#### Refinement

*R*[*F* ^2^ > 2σ(*F* ^2^)] = 0.037
*wR*(*F* ^2^) = 0.095
*S* = 1.05
3500 reflections
315 parameters
H-atom parameters constrained
Δρ_max_ = 0.27 e Å^−3^
Δρ_min_ = −0.18 e Å^−3^

### 

Data collection: *APEX2* (Bruker, 2008 ▶); cell refinement: *SAINT* (Bruker, 2008 ▶); data reduction: *SAINT*; program(s) used to solve structure: *SHELX97* (Sheldrick, 2008 ▶); program(s) used to refine structure: *SHELXL97* (Sheldrick, 2008 ▶); molecular graphics: *X-SEED* (Barbour, 2001 ▶); software used to prepare material for publication: *publCIF* (Westrip, 2009 ▶).

## Supplementary Material

Crystal structure: contains datablocks I, global. DOI: 10.1107/S1600536809030669/xu2575sup1.cif

Structure factors: contains datablocks I. DOI: 10.1107/S1600536809030669/xu2575Isup2.hkl

Additional supplementary materials:  crystallographic information; 3D view; checkCIF report

## Figures and Tables

**Fig. 1.**
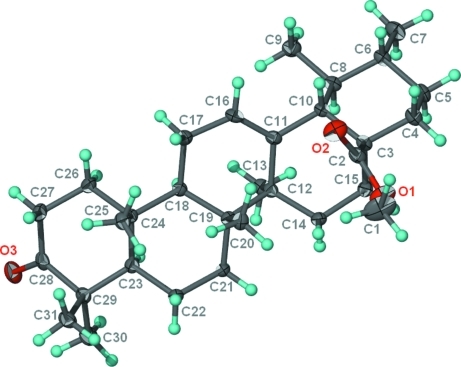
The molecular structure of methyl 3-dehydroursolate. Displacement ellipsoids are drawn at the 70% probability level, and hydrogen atoms are drawn as spheres of arbitrary radius.

## supplementary crystallographic information

### Experimental

The dried leaves of *Primsatomoris malayana* Ridley (Rubiaceae) (2 kg) were extracted with methanol (10 L). The extract was concentrated and then partitioned with petroleum ether, chloroform and ethyl acetate. The chloroform fraction (35 g) was dissolved in methanol and subjected to column chromatography by using Diaion HP-20 with methanol as the eluent to furnish 200 fractions. After confirming that the fractions contained the same material by TLC analysis, the fractions were pooled into 3 sub-fractions. One sub-fraction was purified by using column chromatography on silica gel (chloroform/methanol10:0 → 9:1) to give ursolic acid (5 g), which was identified acid from its NMR and mass spectra.

The ursolic acid was treated with trimethylsilyl diazomethane and pyridinium chlorochromate according to a literature method. The compound was purified by chromatography with a hexane and chloroform system (Ma *et al.*, 2005). Crystals were isolated when the solvent was allowed to evaporate.

### Refinement

The carbon-bound H-atoms were generated geometrically (C—H 0.95–0.99 Å) and were allowed to ride on their parent atoms, with U(H) fixed at 1.2–1.5*U*_eq_(C). Friedel pairs were merged.

### Crystal data

**Table:**

C_31_H_48_O_3_	*F*(000) = 1032
*M**_r_* = 468.69	*D*_x_ = 1.171 Mg m^−^^3^
Orthorhombic, *P*2_1_2_1_2_1_	Mo *K*α radiation, λ = 0.71073 Å
Hall symbol: P 2ac 2ab	Cell parameters from 4540 reflections
*a* = 8.0298 (2) Å	θ = 2.5–28.2°
*b* = 8.4775 (2) Å	µ = 0.07 mm^−^^1^
*c* = 39.0492 (7) Å	*T* = 100 K
*V* = 2658.2 (1) Å^3^	Block, colorless
*Z* = 4	0.25 × 0.15 × 0.10 mm

### Data collection

**Table:**

Bruker SMART APEX diffractometer	3188 reflections with *I* > 2σ(*I*)
Radiation source: fine-focus sealed tube	*R*_int_ = 0.041
graphite	θ_max_ = 27.5°, θ_min_ = 1.0°
ω scans	*h* = −10→10
18588 measured reflections	*k* = −11→10
3500 independent reflections	*l* = −50→49

### Refinement

**Table:**

Refinement on *F*^2^	Secondary atom site location: difference Fourier map
Least-squares matrix: full	Hydrogen site location: inferred from neighbouring sites
*R*[*F*^2^ > 2σ(*F*^2^)] = 0.037	H-atom parameters constrained
*wR*(*F*^2^) = 0.095	*w* = 1/[σ^2^(*F*_o_^2^) + (0.0528*P*)^2^ + 0.4713*P*] where *P* = (*F*_o_^2^ + 2*F*_c_^2^)/3
*S* = 1.05	(Δ/σ)_max_ = 0.001
3500 reflections	Δρ_max_ = 0.27 e Å^−^^3^
315 parameters	Δρ_min_ = −0.18 e Å^−^^3^
0 restraints	Absolute structure: Friedel pairs were merged.
Primary atom site location: structure-invariant direct methods	

### Special details

**Table:**

Geometry. All esds (except the esd in the dihedral angle between two l.s. planes) are estimated using the full covariance matrix. The cell esds are taken into account individually in the estimation of esds in distances, angles and torsion angles; correlations between esds in cell parameters are only used when they are defined by crystal symmetry. An approximate (isotropic) treatment of cell esds is used for estimating esds involving l.s. planes.

### Fractional atomic coordinates and isotropic or equivalent isotropic displacement parameters (Å^2^)

**Table:**

	*x*	*y*	*z*	*U*_iso_*/*U*_eq_	
O1	1.36083 (18)	−0.12139 (18)	0.89475 (4)	0.0258 (3)	
O2	1.10487 (18)	−0.22743 (17)	0.89389 (4)	0.0248 (3)	
O3	0.49772 (19)	0.76407 (17)	0.78616 (4)	0.0240 (3)	
C1	1.4023 (3)	−0.2216 (3)	0.86615 (6)	0.0379 (6)	
H1A	1.5215	−0.2127	0.8612	0.057*	
H1B	1.3381	−0.1889	0.8460	0.057*	
H1C	1.3753	−0.3313	0.8718	0.057*	
C2	1.2031 (2)	−0.1362 (2)	0.90610 (5)	0.0165 (4)	
C3	1.1709 (2)	−0.0328 (2)	0.93751 (4)	0.0140 (4)	
C4	1.2429 (2)	−0.1294 (2)	0.96749 (4)	0.0183 (4)	
H4A	1.2020	−0.2393	0.9659	0.022*	
H4B	1.3659	−0.1319	0.9656	0.022*	
C5	1.1951 (2)	−0.0617 (2)	1.00218 (5)	0.0182 (4)	
H5A	1.2394	−0.1304	1.0206	0.022*	
H5B	1.2454	0.0442	1.0049	0.022*	
C6	1.0070 (2)	−0.0492 (2)	1.00570 (4)	0.0162 (4)	
H6	0.9602	−0.1575	1.0026	0.019*	
C7	0.9619 (3)	0.0053 (3)	1.04214 (5)	0.0223 (4)	
H7A	1.0134	−0.0657	1.0589	0.034*	
H7B	0.8407	0.0034	1.0450	0.034*	
H7C	1.0030	0.1129	1.0458	0.034*	
C8	0.9321 (2)	0.0558 (2)	0.97752 (4)	0.0153 (4)	
H8	0.9783	0.1647	0.9802	0.018*	
C9	0.7425 (2)	0.0651 (3)	0.98027 (5)	0.0200 (4)	
H9A	0.7117	0.1085	1.0026	0.030*	
H9B	0.6950	−0.0408	0.9778	0.030*	
H9C	0.6991	0.1335	0.9621	0.030*	
C10	0.9816 (2)	−0.0071 (2)	0.94123 (4)	0.0137 (4)	
H10	0.9292	−0.1136	0.9390	0.016*	
C11	0.9163 (2)	0.0908 (2)	0.91111 (4)	0.0134 (4)	
C12	1.0129 (2)	0.2364 (2)	0.89869 (4)	0.0130 (3)	
C13	0.9586 (2)	0.3774 (2)	0.92132 (4)	0.0159 (4)	
H13A	0.9966	0.3603	0.9449	0.024*	
H13B	0.8369	0.3864	0.9210	0.024*	
H13C	1.0080	0.4748	0.9124	0.024*	
C14	1.2042 (2)	0.2181 (2)	0.90294 (4)	0.0147 (4)	
H14A	1.2488	0.1635	0.8825	0.018*	
H14B	1.2548	0.3245	0.9037	0.018*	
C15	1.2586 (2)	0.1272 (2)	0.93475 (4)	0.0147 (4)	
H15A	1.2333	0.1907	0.9554	0.018*	
H15B	1.3806	0.1105	0.9339	0.018*	
C16	0.7737 (2)	0.0499 (2)	0.89632 (5)	0.0166 (4)	
H16	0.7174	−0.0390	0.9056	0.020*	
C17	0.6929 (2)	0.1307 (2)	0.86633 (5)	0.0185 (4)	
H17A	0.5766	0.1572	0.8725	0.022*	
H17B	0.6892	0.0562	0.8468	0.022*	
C18	0.7820 (2)	0.2819 (2)	0.85481 (4)	0.0132 (4)	
H18	0.7494	0.3637	0.8720	0.016*	
C19	0.9742 (2)	0.2627 (2)	0.85904 (4)	0.0128 (3)	
C20	1.0371 (2)	0.1194 (2)	0.83823 (5)	0.0162 (4)	
H20A	0.9927	0.1248	0.8149	0.024*	
H20B	0.9994	0.0218	0.8492	0.024*	
H20C	1.1591	0.1209	0.8374	0.024*	
C21	1.0636 (2)	0.4126 (2)	0.84642 (5)	0.0143 (4)	
H21A	1.0470	0.4976	0.8635	0.017*	
H21B	1.1846	0.3910	0.8450	0.017*	
C22	1.0031 (2)	0.4709 (2)	0.81144 (4)	0.0147 (4)	
H22A	1.0630	0.5688	0.8051	0.018*	
H22B	1.0266	0.3903	0.7938	0.018*	
C23	0.8158 (2)	0.5030 (2)	0.81302 (4)	0.0132 (4)	
H23	0.8002	0.5650	0.8346	0.016*	
C24	0.7187 (2)	0.3472 (2)	0.81932 (4)	0.0126 (4)	
C25	0.7322 (2)	0.2282 (2)	0.78945 (5)	0.0169 (4)	
H25A	0.6512	0.2556	0.7717	0.025*	
H25B	0.7091	0.1216	0.7979	0.025*	
H25C	0.8448	0.2318	0.7798	0.025*	
C26	0.5327 (2)	0.3877 (2)	0.82365 (5)	0.0162 (4)	
H26A	0.4680	0.2885	0.8252	0.019*	
H26B	0.5173	0.4457	0.8454	0.019*	
C27	0.4635 (2)	0.4882 (2)	0.79402 (5)	0.0194 (4)	
H27A	0.4628	0.4242	0.7728	0.023*	
H27B	0.3470	0.5180	0.7992	0.023*	
C28	0.5640 (2)	0.6353 (2)	0.78803 (4)	0.0161 (4)	
C29	0.7522 (2)	0.6149 (2)	0.78399 (5)	0.0154 (4)	
C30	0.8357 (3)	0.7772 (2)	0.78758 (5)	0.0221 (4)	
H30A	0.7833	0.8518	0.7717	0.033*	
H30B	0.9545	0.7680	0.7822	0.033*	
H30C	0.8225	0.8152	0.8111	0.033*	
C31	0.7843 (3)	0.5552 (2)	0.74714 (5)	0.0191 (4)	
H31A	0.7515	0.6368	0.7307	0.029*	
H31B	0.7188	0.4595	0.7430	0.029*	
H31C	0.9030	0.5314	0.7443	0.029*	

### Atomic displacement parameters (Å^2^)

**Table:**

	*U*^11^	*U*^22^	*U*^33^	*U*^12^	*U*^13^	*U*^23^
O1	0.0229 (8)	0.0265 (8)	0.0280 (7)	−0.0023 (7)	0.0098 (6)	−0.0121 (7)
O2	0.0256 (8)	0.0206 (7)	0.0282 (7)	−0.0029 (7)	0.0032 (6)	−0.0095 (6)
O3	0.0262 (8)	0.0243 (7)	0.0214 (7)	0.0104 (7)	0.0007 (6)	0.0034 (6)
C1	0.0362 (13)	0.0389 (14)	0.0386 (13)	−0.0035 (12)	0.0176 (11)	−0.0223 (12)
C2	0.0203 (9)	0.0127 (9)	0.0165 (8)	0.0014 (8)	0.0017 (7)	0.0016 (7)
C3	0.0172 (9)	0.0124 (9)	0.0125 (8)	0.0009 (7)	0.0009 (7)	0.0006 (7)
C4	0.0213 (10)	0.0170 (9)	0.0165 (9)	0.0022 (8)	0.0003 (7)	0.0019 (8)
C5	0.0217 (10)	0.0191 (9)	0.0138 (8)	0.0024 (8)	−0.0033 (7)	0.0044 (7)
C6	0.0215 (10)	0.0140 (8)	0.0130 (8)	0.0003 (8)	0.0010 (7)	0.0010 (7)
C7	0.0292 (11)	0.0238 (10)	0.0139 (9)	−0.0002 (9)	0.0005 (8)	0.0007 (8)
C8	0.0185 (10)	0.0139 (9)	0.0135 (8)	−0.0003 (8)	0.0018 (7)	0.0005 (7)
C9	0.0200 (10)	0.0241 (10)	0.0158 (9)	−0.0001 (8)	0.0033 (7)	−0.0005 (8)
C10	0.0155 (9)	0.0126 (8)	0.0130 (8)	−0.0005 (7)	0.0007 (7)	0.0007 (7)
C11	0.0154 (9)	0.0124 (9)	0.0123 (8)	0.0009 (7)	0.0033 (7)	0.0001 (7)
C12	0.0145 (8)	0.0115 (8)	0.0130 (8)	−0.0004 (7)	−0.0003 (7)	0.0003 (7)
C13	0.0208 (10)	0.0136 (9)	0.0134 (8)	−0.0010 (8)	−0.0003 (7)	−0.0014 (7)
C14	0.0133 (9)	0.0169 (9)	0.0140 (8)	−0.0026 (8)	0.0005 (7)	0.0020 (7)
C15	0.0145 (9)	0.0152 (9)	0.0144 (8)	−0.0008 (8)	−0.0011 (7)	−0.0006 (7)
C16	0.0201 (9)	0.0143 (9)	0.0154 (8)	−0.0023 (8)	0.0021 (7)	0.0033 (7)
C17	0.0156 (9)	0.0225 (10)	0.0172 (9)	−0.0046 (8)	−0.0018 (7)	0.0043 (8)
C18	0.0127 (8)	0.0150 (9)	0.0119 (8)	−0.0002 (7)	0.0003 (6)	0.0004 (7)
C19	0.0110 (8)	0.0143 (8)	0.0131 (8)	−0.0006 (7)	0.0008 (6)	0.0005 (7)
C20	0.0162 (9)	0.0180 (9)	0.0142 (8)	0.0025 (8)	0.0001 (7)	−0.0026 (7)
C21	0.0109 (9)	0.0165 (9)	0.0153 (8)	−0.0017 (7)	−0.0006 (7)	0.0034 (7)
C22	0.0122 (9)	0.0178 (9)	0.0142 (8)	−0.0010 (8)	0.0013 (7)	0.0028 (7)
C23	0.0131 (9)	0.0145 (9)	0.0119 (8)	0.0005 (7)	0.0000 (6)	−0.0005 (7)
C24	0.0110 (8)	0.0149 (9)	0.0117 (8)	0.0008 (7)	0.0003 (6)	0.0007 (7)
C25	0.0185 (9)	0.0172 (9)	0.0151 (8)	0.0004 (8)	−0.0016 (7)	−0.0007 (7)
C26	0.0127 (9)	0.0210 (10)	0.0150 (8)	−0.0006 (8)	0.0004 (7)	0.0023 (8)
C27	0.0124 (9)	0.0268 (11)	0.0191 (9)	0.0028 (8)	−0.0007 (7)	0.0021 (8)
C28	0.0181 (9)	0.0228 (10)	0.0074 (7)	0.0048 (8)	−0.0010 (6)	0.0000 (7)
C29	0.0168 (9)	0.0138 (9)	0.0155 (8)	0.0009 (8)	−0.0009 (7)	0.0023 (7)
C30	0.0251 (10)	0.0150 (9)	0.0262 (10)	−0.0010 (9)	−0.0042 (8)	0.0030 (8)
C31	0.0222 (10)	0.0210 (10)	0.0140 (8)	0.0011 (9)	0.0020 (7)	0.0031 (8)

### Geometric parameters (Å, °)

**Table:**

O1—C2	1.348 (2)	C15—H15B	0.9900
O1—C1	1.442 (2)	C16—C17	1.504 (3)
O2—C2	1.203 (2)	C16—H16	0.9500
O3—C28	1.217 (2)	C17—C18	1.535 (3)
C1—H1A	0.9800	C17—H17A	0.9900
C1—H1B	0.9800	C17—H17B	0.9900
C1—H1C	0.9800	C18—C19	1.560 (2)
C2—C3	1.529 (3)	C18—C24	1.577 (2)
C3—C15	1.532 (3)	C18—H18	1.0000
C3—C4	1.542 (3)	C19—C21	1.540 (3)
C3—C10	1.543 (3)	C19—C20	1.546 (3)
C4—C5	1.521 (3)	C20—H20A	0.9800
C4—H4A	0.9900	C20—H20B	0.9800
C4—H4B	0.9900	C20—H20C	0.9800
C5—C6	1.520 (3)	C21—C22	1.532 (2)
C5—H5A	0.9900	C21—H21A	0.9900
C5—H5B	0.9900	C21—H21B	0.9900
C6—C8	1.538 (3)	C22—C23	1.530 (3)
C6—C7	1.539 (2)	C22—H22A	0.9900
C6—H6	1.0000	C22—H22B	0.9900
C7—H7A	0.9800	C23—C24	1.554 (3)
C7—H7B	0.9800	C23—C29	1.563 (3)
C7—H7C	0.9800	C23—H23	1.0000
C8—C9	1.529 (3)	C24—C26	1.542 (3)
C8—C10	1.565 (2)	C24—C25	1.546 (2)
C8—H8	1.0000	C25—H25A	0.9800
C9—H9A	0.9800	C25—H25B	0.9800
C9—H9B	0.9800	C25—H25C	0.9800
C9—H9C	0.9800	C26—C27	1.540 (3)
C10—C11	1.532 (2)	C26—H26A	0.9900
C10—H10	1.0000	C26—H26B	0.9900
C11—C16	1.328 (3)	C27—C28	1.504 (3)
C11—C12	1.536 (3)	C27—H27A	0.9900
C12—C13	1.550 (3)	C27—H27B	0.9900
C12—C14	1.553 (2)	C28—C29	1.529 (3)
C12—C19	1.595 (2)	C29—C30	1.537 (3)
C13—H13A	0.9800	C29—C31	1.547 (3)
C13—H13B	0.9800	C30—H30A	0.9800
C13—H13C	0.9800	C30—H30B	0.9800
C14—C15	1.525 (2)	C30—H30C	0.9800
C14—H14A	0.9900	C31—H31A	0.9800
C14—H14B	0.9900	C31—H31B	0.9800
C15—H15A	0.9900	C31—H31C	0.9800
			
C2—O1—C1	114.64 (17)	C16—C17—C18	114.04 (16)
O1—C1—H1A	109.5	C16—C17—H17A	108.7
O1—C1—H1B	109.5	C18—C17—H17A	108.7
H1A—C1—H1B	109.5	C16—C17—H17B	108.7
O1—C1—H1C	109.5	C18—C17—H17B	108.7
H1A—C1—H1C	109.5	H17A—C17—H17B	107.6
H1B—C1—H1C	109.5	C17—C18—C19	110.08 (15)
O2—C2—O1	123.04 (18)	C17—C18—C24	113.61 (15)
O2—C2—C3	125.15 (18)	C19—C18—C24	116.65 (14)
O1—C2—C3	111.67 (16)	C17—C18—H18	105.1
C2—C3—C15	111.93 (15)	C19—C18—H18	105.1
C2—C3—C4	103.95 (15)	C24—C18—H18	105.1
C15—C3—C4	110.59 (15)	C21—C19—C20	109.13 (14)
C2—C3—C10	108.83 (15)	C21—C19—C18	109.96 (15)
C15—C3—C10	109.55 (15)	C20—C19—C18	110.44 (15)
C4—C3—C10	111.90 (15)	C21—C19—C12	109.60 (14)
C5—C4—C3	112.40 (15)	C20—C19—C12	109.65 (14)
C5—C4—H4A	109.1	C18—C19—C12	108.04 (13)
C3—C4—H4A	109.1	C19—C20—H20A	109.5
C5—C4—H4B	109.1	C19—C20—H20B	109.5
C3—C4—H4B	109.1	H20A—C20—H20B	109.5
H4A—C4—H4B	107.9	C19—C20—H20C	109.5
C6—C5—C4	110.98 (16)	H20A—C20—H20C	109.5
C6—C5—H5A	109.4	H20B—C20—H20C	109.5
C4—C5—H5A	109.4	C22—C21—C19	113.81 (15)
C6—C5—H5B	109.4	C22—C21—H21A	108.8
C4—C5—H5B	109.4	C19—C21—H21A	108.8
H5A—C5—H5B	108.0	C22—C21—H21B	108.8
C5—C6—C8	111.37 (15)	C19—C21—H21B	108.8
C5—C6—C7	109.76 (16)	H21A—C21—H21B	107.7
C8—C6—C7	113.34 (16)	C23—C22—C21	109.47 (15)
C5—C6—H6	107.4	C23—C22—H22A	109.8
C8—C6—H6	107.4	C21—C22—H22A	109.8
C7—C6—H6	107.4	C23—C22—H22B	109.8
C6—C7—H7A	109.5	C21—C22—H22B	109.8
C6—C7—H7B	109.5	H22A—C22—H22B	108.2
H7A—C7—H7B	109.5	C22—C23—C24	110.40 (15)
C6—C7—H7C	109.5	C22—C23—C29	113.55 (15)
H7A—C7—H7C	109.5	C24—C23—C29	117.82 (15)
H7B—C7—H7C	109.5	C22—C23—H23	104.5
C9—C8—C6	111.65 (16)	C24—C23—H23	104.5
C9—C8—C10	109.50 (15)	C29—C23—H23	104.5
C6—C8—C10	110.56 (15)	C26—C24—C25	107.21 (15)
C9—C8—H8	108.3	C26—C24—C23	108.31 (15)
C6—C8—H8	108.3	C25—C24—C23	113.59 (14)
C10—C8—H8	108.3	C26—C24—C18	107.13 (14)
C8—C9—H9A	109.5	C25—C24—C18	114.27 (15)
C8—C9—H9B	109.5	C23—C24—C18	106.02 (14)
H9A—C9—H9B	109.5	C24—C25—H25A	109.5
C8—C9—H9C	109.5	C24—C25—H25B	109.5
H9A—C9—H9C	109.5	H25A—C25—H25B	109.5
H9B—C9—H9C	109.5	C24—C25—H25C	109.5
C11—C10—C3	109.96 (15)	H25A—C25—H25C	109.5
C11—C10—C8	115.04 (15)	H25B—C25—H25C	109.5
C3—C10—C8	112.55 (15)	C27—C26—C24	112.97 (15)
C11—C10—H10	106.2	C27—C26—H26A	109.0
C3—C10—H10	106.2	C24—C26—H26A	109.0
C8—C10—H10	106.2	C27—C26—H26B	109.0
C16—C11—C10	119.15 (17)	C24—C26—H26B	109.0
C16—C11—C12	120.51 (17)	H26A—C26—H26B	107.8
C10—C11—C12	120.32 (16)	C28—C27—C26	112.43 (16)
C11—C12—C13	107.33 (14)	C28—C27—H27A	109.1
C11—C12—C14	112.66 (15)	C26—C27—H27A	109.1
C13—C12—C14	107.13 (15)	C28—C27—H27B	109.1
C11—C12—C19	108.71 (14)	C26—C27—H27B	109.1
C13—C12—C19	113.01 (15)	H27A—C27—H27B	107.8
C14—C12—C19	108.08 (14)	O3—C28—C27	121.22 (17)
C12—C13—H13A	109.5	O3—C28—C29	121.86 (19)
C12—C13—H13B	109.5	C27—C28—C29	116.92 (17)
H13A—C13—H13B	109.5	C28—C29—C30	108.67 (16)
C12—C13—H13C	109.5	C28—C29—C31	107.33 (15)
H13A—C13—H13C	109.5	C30—C29—C31	107.77 (16)
H13B—C13—H13C	109.5	C28—C29—C23	108.44 (15)
C15—C14—C12	114.89 (15)	C30—C29—C23	109.54 (15)
C15—C14—H14A	108.5	C31—C29—C23	114.94 (15)
C12—C14—H14A	108.5	C29—C30—H30A	109.5
C15—C14—H14B	108.5	C29—C30—H30B	109.5
C12—C14—H14B	108.5	H30A—C30—H30B	109.5
H14A—C14—H14B	107.5	C29—C30—H30C	109.5
C14—C15—C3	111.87 (15)	H30A—C30—H30C	109.5
C14—C15—H15A	109.2	H30B—C30—H30C	109.5
C3—C15—H15A	109.2	C29—C31—H31A	109.5
C14—C15—H15B	109.2	C29—C31—H31B	109.5
C3—C15—H15B	109.2	H31A—C31—H31B	109.5
H15A—C15—H15B	107.9	C29—C31—H31C	109.5
C11—C16—C17	126.25 (18)	H31A—C31—H31C	109.5
C11—C16—H16	116.9	H31B—C31—H31C	109.5
C17—C16—H16	116.9		
			
C1—O1—C2—O2	1.3 (3)	C24—C18—C19—C21	46.9 (2)
C1—O1—C2—C3	177.10 (18)	C17—C18—C19—C20	57.77 (19)
O2—C2—C3—C15	−145.83 (19)	C24—C18—C19—C20	−73.6 (2)
O1—C2—C3—C15	38.5 (2)	C17—C18—C19—C12	−62.16 (18)
O2—C2—C3—C4	94.8 (2)	C24—C18—C19—C12	166.51 (15)
O1—C2—C3—C4	−80.92 (19)	C11—C12—C19—C21	177.46 (14)
O2—C2—C3—C10	−24.6 (3)	C13—C12—C19—C21	58.42 (19)
O1—C2—C3—C10	159.69 (16)	C14—C12—C19—C21	−59.95 (18)
C2—C3—C4—C5	−169.25 (16)	C11—C12—C19—C20	−62.76 (18)
C15—C3—C4—C5	70.5 (2)	C13—C12—C19—C20	178.20 (15)
C10—C3—C4—C5	−52.0 (2)	C14—C12—C19—C20	59.82 (19)
C3—C4—C5—C6	56.4 (2)	C11—C12—C19—C18	57.66 (18)
C4—C5—C6—C8	−58.8 (2)	C13—C12—C19—C18	−61.39 (19)
C4—C5—C6—C7	174.87 (15)	C14—C12—C19—C18	−179.76 (15)
C5—C6—C8—C9	178.32 (17)	C20—C19—C21—C22	73.64 (19)
C7—C6—C8—C9	−57.3 (2)	C18—C19—C21—C22	−47.6 (2)
C5—C6—C8—C10	56.1 (2)	C12—C19—C21—C22	−166.27 (15)
C7—C6—C8—C10	−179.51 (16)	C19—C21—C22—C23	57.9 (2)
C2—C3—C10—C11	−66.30 (19)	C21—C22—C23—C24	−65.05 (18)
C15—C3—C10—C11	56.37 (19)	C21—C22—C23—C29	160.11 (15)
C4—C3—C10—C11	179.39 (15)	C22—C23—C24—C26	175.43 (14)
C2—C3—C10—C8	164.02 (15)	C29—C23—C24—C26	−51.87 (19)
C15—C3—C10—C8	−73.31 (18)	C22—C23—C24—C25	−65.58 (19)
C4—C3—C10—C8	49.7 (2)	C29—C23—C24—C25	67.1 (2)
C9—C8—C10—C11	57.7 (2)	C22—C23—C24—C18	60.73 (17)
C6—C8—C10—C11	−178.85 (16)	C29—C23—C24—C18	−166.57 (15)
C9—C8—C10—C3	−175.27 (16)	C17—C18—C24—C26	61.65 (19)
C6—C8—C10—C3	−51.8 (2)	C19—C18—C24—C26	−168.67 (16)
C3—C10—C11—C16	137.50 (18)	C17—C18—C24—C25	−56.9 (2)
C8—C10—C11—C16	−94.2 (2)	C19—C18—C24—C25	72.7 (2)
C3—C10—C11—C12	−43.7 (2)	C17—C18—C24—C23	177.16 (15)
C8—C10—C11—C12	84.6 (2)	C19—C18—C24—C23	−53.2 (2)
C16—C11—C12—C13	93.9 (2)	C25—C24—C26—C27	−70.3 (2)
C10—C11—C12—C13	−84.86 (19)	C23—C24—C26—C27	52.61 (19)
C16—C11—C12—C14	−148.42 (17)	C18—C24—C26—C27	166.58 (15)
C10—C11—C12—C14	32.8 (2)	C24—C26—C27—C28	−53.9 (2)
C16—C11—C12—C19	−28.6 (2)	C26—C27—C28—O3	−129.36 (18)
C10—C11—C12—C19	152.60 (15)	C26—C27—C28—C29	51.4 (2)
C11—C12—C14—C15	−35.7 (2)	O3—C28—C29—C30	15.4 (2)
C13—C12—C14—C15	82.15 (19)	C27—C28—C29—C30	−165.39 (16)
C19—C12—C14—C15	−155.78 (15)	O3—C28—C29—C31	−100.9 (2)
C12—C14—C15—C3	52.3 (2)	C27—C28—C29—C31	78.33 (19)
C2—C3—C15—C14	58.1 (2)	O3—C28—C29—C23	134.38 (18)
C4—C3—C15—C14	173.52 (15)	C27—C28—C29—C23	−46.4 (2)
C10—C3—C15—C14	−62.68 (19)	C22—C23—C29—C28	179.09 (15)
C10—C11—C16—C17	−179.08 (17)	C24—C23—C29—C28	47.8 (2)
C12—C11—C16—C17	2.2 (3)	C22—C23—C29—C30	−62.5 (2)
C11—C16—C17—C18	−5.3 (3)	C24—C23—C29—C30	166.24 (16)
C16—C17—C18—C19	35.7 (2)	C22—C23—C29—C31	59.0 (2)
C16—C17—C18—C24	168.65 (15)	C24—C23—C29—C31	−72.3 (2)
C17—C18—C19—C21	178.27 (14)		
